# Preventing Infections by Encapsulated Bacteria Through Vaccine Prophylaxis in Inflammatory Bowel Disease

**DOI:** 10.3389/fimmu.2020.00485

**Published:** 2020-03-23

**Authors:** Marco Vincenzo Lenti, Caterina Mengoli, Marta Vernero, Nicola Aronico, Laura Conti, Federica Borrelli de Andreis, Sara Cococcia, Antonio Di Sabatino

**Affiliations:** ^1^First Department of Internal Medicine, San Matteo Hospital Foundation, University of Pavia, Pavia, Italy; ^2^Medical-Surgical Department of Clinical Sciences and Translational Medicine, Sapienza University of Rome, Rome, Italy

**Keywords:** Crohn's disease, hyposplenism, opportunistic infections, ulcerative colitis, vaccination strategy

## Abstract

Inflammatory bowel disease (IBD), which comprises ulcerative colitis and Crohn's disease, is an immune-mediated, chronic-relapsing, disabling disorder which is associated with increased mortality and poor patients' quality of life. Patients with IBD are at increased risk of infections for many reasons. In fact, IBD often requires a lifelong immunosuppressive and/or biologic therapy, both commonly associated with respiratory and opportunistic infections, but also gastrointestinal, urinary tract infections, and sepsis. Moreover, impaired spleen function has been found in a considerable proportion of IBD patients, further increasing the risk of developing infections sustained by encapsulated bacteria, such as *S. pneumoniae, H. influenzae*, and *N. meningitidis*. Finally, comorbidities and surgery represent additional risk factors for these patients. Despite the availability of vaccinations against the most common serotypes of encapsulated bacteria, uncertainties still exist regarding a proper vaccination strategy and the actual effectiveness of vaccinations in this particular setting. Aim of this narrative review is to focus on the broad topic of vaccinations against encapsulated bacteria in IBD patients, discussing the clinical impact of infections, predisposing factors, vaccinations strategies, and unmet research and clinical needs.

## Introduction

Inflammatory bowel disease (IBD), which includes Crohn's disease (CD) and ulcerative colitis (UC), is an immune-mediated disease affecting both the colon and the small intestine (1, 2). Due to the chronic immune-mediated inflammation of the bowel, IBD often requires immunosuppressive therapies, such as corticosteroids, thiopurines, methotrexate (MTX), anti-tumor necrosis factor (TNF) alpha agents or other biological drugs. Although IBD patients are not routinely considered as immunodeficient, if not secondary to immunosuppressive therapies, there is evidence of impaired immune responses in IBD (3). In fact, we know from genome wide association studies that there is an aberrant immune response in IBD, involving both innate and adaptive immune response loci (4). Nevertheless, recent evidence shows that reduction of microbiome diversity is typical of IBD, and this could be another factor underlying immune impairment in these patients (5). As a result of the exogenous and the endogenous immunosuppression, these patients are at a higher risk of infections, especially those from encapsulated bacteria (6, 7). This could also be the consequence of impaired spleen function (also known as hyposplenism), that is quite common in this population (8, 9). Indeed, the risk of invasive pneumococcus infection is increased 3-fold during the first year from IBD diagnosis, and it is still high during the following years, and this is true regardless of immunosuppressive therapy. This is the main reason why encapsulated bacteria vaccination (especially anti pneumococcal vaccine) is strongly recommended after IBD diagnosis, ideally at least 2 weeks before starting any immunosuppressive therapy (10).

Despite the possibility to prevent most of these infections through vaccines, vaccination rate among IBD patients is still very low (11, 12). It is advisable to check immunization state of the patients and proceed to vaccination, in order to be able to initiate immunosuppressive therapies as soon as possible, when needed. As said, immunization rate among IBD patients is still low and this is due to both lack of knowledge and proposal among gastroenterologists and primary care physicians (5, 10).

On these bases, the aim of this narrative review is to clarify the reasons why IBD patients should be vaccinated, especially against encapsulated bacteria. Furthermore, we aim to summarize all the current indications to immunization among IBD patients stressing the need to routinely vaccinate these patients at the time of diagnosis.

## Clinical Impact of Infections by Encapsulated Bacteria in IBD

Patients with IBD are at increased risk for infections, many of which are potentially preventable through the use of available vaccines (11). Infections are one of the most frequent comorbid conditions in IBD in which there is also an increased likelihood of developing severe manifestations from encapsulated microorganisms, including *S. pneumoniae, H. influenzae*, and *N. meningitidis*. Impairment of the innate immune response against infectious agents observed in patients with IBD (13) seems to be the most important factor affecting susceptibility to infections. Also, treatment with immunosuppressive agents and hyposplenism (8, 14) are additional factors.

In 2015, the mean incidence rate for invasive meningococcal disease in the general population in Europe was 0.6 per 100,000 individuals, higher among infants and young children (15). Invasive pneumococcal disease was reported in 5.5 per 100,000 individuals, higher among infants and adults aged 65 years or over (15). Finally, invasive *H. influenzae* disease was reported in 0.7 per 100,000 individuals, with peaks among infants under 1 year and in the elderly (16). An Italian study compared the national surveillance system with recorded hospitalizations occurring between 2007 and 2016 for encapsulated bacterial infections, identifying 12,671 hospital discharges with a diagnosis consistent with infection by *S. pneumoniae, H. influenzae*, and *N. meningitidis*. The most frequent bacterial agent found in this study was *S. pneumoniae* (9,430, 74.4%), followed by *N. meningitidis* (2,067, 16.3%), and by *H. influenzae* (1,174, 9.3%) (17).

A few studies explored invasive *S. pneumoniae* infections in IBD patients, who are at increased risk of death during hospitalization (18, 19). Instead, data regarding the incidence rate of infections due to *H. influenzae* and *N. meningitidis* are still scant (20). In line with other countries, in the US the most common etiologic agent of community acquired pneumonia is *S. pneumoniae*. Long et al. conducted a large retrospective study to define the risk of pneumonia in IBD patients and how immunosuppressive treatments affect this risk (21). The study included 50,932 CD patients, 56,403 UC patients, and 1,269 with unspecified IBD matched with 434,416 individuals without IBD. IBD patients turned out to have one and a half times higher rate of pneumonia (HR 1.54, 95% CI 1.49–1.60) compared to age-matched controls without IBD, with an increased risk in both CD (HR 1.71, 95% CI 1.62–1.80) and UC (HR 1.41, 95% CI 1.34–1.48). Unfortunately, this study did not differentiate vaccine preventable pneumonia from others, hence it is difficult to ascertain the impact of vaccination.

Another population-based study aiming to investigate the risk of invasive pneumococcal disease (IPD) before and after IBD diagnosis was recently conducted in Denmark (7). This study included 74,156 IBD patients, 1,482,363 non-IBD controls, and matched individuals from the general population. The study found 277 IPD cases among IBD patients (0.37%) and 3,984 in controls (0.27%), proving a 2-fold higher risk of IPD in CD patients than controls (HR 1.99; 95% CI, 1.59–2.49), whereas a 1.5-fold higher risk was found in UC patients (HR 1.46; 95% CI, 1.25–1.69). Furthermore, the study demonstrated that IBD patients had an increased risk of IPD, both before and after IBD diagnosis, suggesting that it is likely related to the underlying altered immune response and, in contrast with other studies, not associated with the use of immunosuppressors or immunomodulators. A major limit of this study was the lack of pneumococcal vaccination data over the study period.

A further study from the US showed that the prevalence of *S. pneumoniae* infection in IBD patients hospitalized for pneumonia was 82.6 per 100,000, while only 69.2 per 100,000 for the control population. Thirteen deaths occurred among IBD patients. Moreover, during the 6-year study period, the prevalence of *H. influenzae* pneumonia cases among IBD patients was 19.2 per 100,000, compared with 14 per 100,000 in the control population, with in-hospital five deaths (22).

Meningococcal infections are endemic in Western countries and infections caused by *N. meningitidis* can evolve into a disease with high mortality, if not recognized and promptly treated. Meningococcal infections have only been reported in small series of IBD patients (23, 24).

Table 1 reports the main results of studies exploring encapsulated bacterial infections in IBD patients.

**Table 1:** Summary of the main studies focusing on encapsulated bacterial infections in inflammatory bowel disease.

**References**	**Country**	**Study design**	**Patients, *n***	**Infection type**	**Infection risk**
Long et al. (21)	US	Retrospective cohort study Nested case-control study	50,932 CD 56,403 UC 1,269 unspecified IBD 434,416 Hc	Pneumonia	HR 1.71, 95% CI 1.62–1.80 (CD) HR 1.41, 95% CI 1.34–1.48 (UC)
Kantsø et al. (7)	Denmark	Population-based cohort study	22,098 CD 52,058 UC 1,482,363 Hc	IPD	HR 1.99; 95% CI, 1.59–2.49 (CD) HR, 1.46; 95% CI, 1.25–1.69 (UC)
Stobaugh et al. (22)	US	Cross-sectional study Nationwide inpatient sample	48,087,002 inpatient discharge visits	Pneumonia *S. pneumoniae H. influenzae*	HR 1.08; 95% CI, 0.99–1.17 (CD) HR 0.93; 95% CI, 0.82–1.06 (UC) HR 1.28; 95% CI, 1.06–1.54 (CD) HR 1.42; 95% CI, 1.13–1.79 (UC)

*CD, Crohn disease; Hc, Health controls; IPD invasive pneumococcal infection; UC, ulcerative colitis*.

## Predisposing Factors

### Hyposplenism

Spleen function in health and disease has drawn progressive attention over the last decades, especially in relation to the gastrointestinal tract (9, 14, 25). The spleen structure consists of three interrelated compartments—the red pulp, the white pulp, and the marginal zone. Among other functions—including phagocytic filter, antibodies production and maintenance of immunological tolerance—the spleen plays a crucial role in the clearance of encapsulated bacteria (25). In fact, IgM memory B cells, which are a unique B-cell population of the marginal zone of the spleen are responsible for producing pentameric IgM antibodies which are necessary to facilitate phagocytosis of these bacteria, either directly or through complement deposition on the capsule (26, 27). IgM memory B cells, however, can only be produced if spleen function is unaltered, and are thus diminished in patients with common variable immunodeficiency, congenital or acquired asplenia, and in the elderly (25). A variety of diseases—including IBD and other gastrointestinal, hematologic, hepatic, autoimmune, infectious, congenital diseases—may present splenic abnormalities that can vary from mild hyposplenism to frank splenic atrophy. In clinical practice, the assessment of spleen function can be easily performed through counting of pitted red cells with an interference contrast microscope equipped with Nomarski optic, with an upper limit of normal of 4%. Pitted red cells are erythrocytes with an irregular surface which are normally cleared by a normal-functioning spleen (28).

IBD patients, who already face an increased risk of infection due to their disease, due to hyposplenism may also be less likely to clear an infection driven by encapsulated bacteria (29, 30), which can be avoided through the use of vaccines, especially conjugated vaccines (31, 32). This is the reason why, in these patients, hyposlenism should be appropriately assessed. The relation between hyposplenism and IBD was firstly described ~40 years ago (33, 34), and it was related to disease activity, especially in UC (35). Even if the fine bases of hyposplenism in IBD are not yet fully understood, depletion or impairment of enteric lymphocytes seem to be implied. Also, depletion of IgM memory B cells was shown in patients with both CD and UC, with an inverse relation with pitted red cells, which strongly suggests that IgM memory B cells depletion is directly related to the degree of splenic impairment (8). More studies focusing on hyposplenism in IBD are eagerly awaited.

### Immunosuppressive Therapy

The first use of corticosteroids for IBD dates back to 1955, when Truelove and Witts demonstrated their efficacy in inducing remission in UC (36). Soon after, in 1979, Summers et al. showed the same effect in CD (37). Since then, these drugs have been the milestone of moderate to severe IBD medical treatment. However, patients usually relapse shortly after tapering and require up to 20–30 mg of steroids daily to maintain remission. This condition is called steroid resistance and indicates the need for immunosuppressive therapies including thiopurines and MTX (38, 39). 6-mercaptopurine and its prodrug azathioprine exert an immune modifier function due to their antimetabolite activity that reduces cell proliferation. This peculiar characteristic could be a double-edged sword, as on one hand they have been shown to be effective in both inducing (40) and maintaining (41) remission in IBD, but, on the other hand, they could lead to an immune impairment with subsequent higher risk of hematologic and non-hematologic neoplasia and infections, especially among elderly patients. MTX is an antiproliferative molecule inhibiting dihydrofolate reductase used at high dose as chemotherapy and at low dose as immunomodulator for chronic inflammatory diseases, including rheumatoid arthritis (42) and IBD (43). Notwithstanding its efficacy, its anti-inflammatory effect is still not fully understood but may be due to antiproliferative effects on leukocytes and decreased inflammatory molecule production (44). At present, MTX is used in CD patients, but its use in UC is still controversial due to the lack of evidence on its efficacy (45). Nevertheless, immunosuppressive therapies are often used in combination with biological therapies (especially with anti-TNF drugs) in order to reduce their immunogenicity, raising concerns about possible side effects, especially infections and malignancy (45). Indeed, infections represent one of the leading causes of death in individuals with IBD (46).

IBD patients have a higher morbidity and mortality rate for infectious complications compared to the general population (19). However, most of the current knowledge in this regard derives from other autoimmune conditions requiring prolonged and intensive immunosuppressive therapies. For example, a few studies demonstrated that pneumonia is one of the most frequent causes of morbidity and mortality in patients with rheumatoid arthritis (47, 48). Also, a few cases of fulminant infections have been reported among IBD, Still's disease, and patients with rheumatoid arthritis undergoing immunosuppressive therapies (11).

According to European Crohn's and Colitis Organization, a dose of at least 2 mg/kg of intravenous steroids or at least 20 mg of oral prednisone for more than 2 weeks is a risk factor for infectious diseases together with thiopurines, MTX, and calcineurin inhibitors (5). According to the literature, corticosteroid administration is clearly associated with serious infections in a dose-dependent fashion (49, 50).

According to the study by Longo et al. an increased risk of infection among patients on steroids (OR 1.91 95%CI 1.72–2.12) and on thiopurines (OR 1.13 95% IC 1.00–1.27) (21) was noticed. These findings do not differ from those of patients with rheumatoid arthritis, highlighting the risk for pneumonia attributable to the use of corticosteroids (47, 51), but no additional risk due to other disease modifying antirheumatic drugs, especially MTX (52). Moreover, as both corticosteroid use *per se* and infections have been associated with an excess of mortality, preventive strategies should represent a priority in the immediate future (22, 53). Indeed, guidelines promoted by the European Crohn's and Colitis Organization suggest vaccination against encapsulated bacteria (particularly *S. pneumoniae*) before starting immunosuppressive therapies (5). Nonetheless, there is evidence that pneumococcal vaccination is poor among IBD patients (11), due to both unawareness and intentional lack of adherence.

Finally, there is lack of data regarding specifically *H. influenzae* and *N. Meningitidis* among IBD patients, especially those undergoing immunosuppressive therapy.

Besides medical therapy, IBD patients needing immunosuppressive drugs have a more severe illness, so the highest prevalence of infections could be related to disease activity itself (21), and to hyposplenism (8, 25).

### Biological Therapies

TNF is a proinflammatory cytokine involved in a variety of pathways including innate and adaptive immunity, inflammation response and cell death regulation (54). Even though the exact etiology is still unknown, high concentrations of this cytokine are believed to play a key role in the pathophysiology of IBD, causing chronic inflammation and Th-1 exacerbation in a high percentage of IBD patients (55–57). Therefore, TNF alpha-inhibitors, including infliximab, adalimumab, certolizumab, and golimumab, which have been used as effective drugs for these ailments, still raise concerns about their safety due to the pathways they interfere with. Different studies were conducted to evaluate if there was a higher risk of severe infection in those patients treated with anti-TNF therapy, showing a sizeable increase, up to 2-fold, which correlates with the dosage and the association with other immunosuppressive therapies (58–61). Alongside with those evidences, since *S. pneumoniae* is the first cause of community-acquired pneumonia (62), immunization in IBD patients receiving anti-TNF is recommended by both European and American guidelines (5, 18). However, the response to the 23-valent pneumococcal polysaccharide vaccine in patients receiving anti-TNF therapy, has been shown to be significantly lower when mirrored with the healthy population (63–65), and thus the vaccine should be administrated before starting an immunosuppressive therapy, whenever possible.

To face the lack of selectiveness, new drugs targeting gut-specific receptors have been studied. Vedolizumab is a humanized IgG1 monoclonal antibody binding to the α4β7 integrin, selectively dampening the lymphocyte activity in the gut thanks to the lack of affinity to α4 (66–69). Due to its different mechanism of action, vedolizumab seems to be safer than anti-TNF drugs showing lower risk of infections in both UC and CD patients (66, 70–73).

Ustekinumab is a fully human immunoglobulin G1k monoclonal antibody that selectively target the IL12/IL23 p40 subunit, interfering with the regulatory cytokines involved in inflammatory and immune response, natural killer cells activation and effector cytokine production (e.g., TNF, IL-17, IL-22) (74, 75). Available data on this drug suggest no correlation between ustekinumab therapy and any kind of infections in different cohorts of patients (CD or psoriasis), with a higher incidence amongst CD patients. However, incidence was comparable between ustekinumab and placebo-treated patients, with no apparent dose-effect correlation, suggesting that the higher rates saw in this cohort were the results of the severity of disease activity (76–80).

### Comorbidities

Comorbidity is one of the major factors contributing to patients' complexity, leading to a more difficult therapeutic approach, especially when it is associated with frailty. Physicians often have to face with multimorbid patients and this could be due to the spreading of unhealthy lifestyle and to the longer life expectancy (81). Moreover, comorbidity worsens the prognosis of IBD *per se*, increasing the likelihood of drug-to-drug interaction. Kariyawasam et al. demonstrated that comorbidities, rather than age itself, are the major risk factors for a worse outcome and for a higher need for immunosuppressive drugs (82).

Similarly to what is reported in the general population, incidence of encapsulated bacterial infections (*S. pneumoniae* in most cases) is higher in elderly IBD patients and in individuals with comorbidities. For instance, in a recent study investigating hospitalization for infectious disease in the first year from IBD diagnosis, it was demonstrated that the presence of comorbid conditions is an independent risk factor for this outcome (OR 2.32; 95% CI, 1.05–5.13) (83).

Particularly, alcoholism, organic brain disease, diabetes mellitus and chronic lung disease are considered major independent risk factors for infectious diseases among IBD patients, confirming what was previously reported about patients with rheumatoid arthritis (5, 84).

The importance of diabetes as an additional risk factor for *S. pneumoniae* pneumonia (HR 1.92 95% IC1.84–1.99) and for death (HR 1.67 95% IC 1.45–1.92) has been reported. Moreover, chronic obstructive pulmonary disease is a risk factor for infections, especially those affecting the respiratory tract. According to this study, besides comorbidity, older age is another important co-factor contributing to the increased risk of bacterial infections. Particularly, among elderly individuals, the risk was significantly higher compared to younger patients (age limit 30 years), with the highest absolute risk among individuals aged 61–64 years (21). Moreover, malnutrition, total parenteral nutrition, and bowel surgery were independently associated with infectious-related hospitalization (19). Indeed, malnutrition is a rather common condition in these patients, resulting from inadequate food intake (due to gastrointestinal symptoms), malabsorption, surgery, short bowel syndrome, and drug interactions (85).

Interestingly, recent findings stressed the importance of the chronic use of proton pumps inhibitors as a risk factor for community acquired pneumonia. All comorbidities that require the use of this class of medication may lead to a higher risk of *S. pneumoniae* infection (86).

### Surgery

Surgery represents a risk factor *per se* for infections, especially if performed in a non-elective fashion as it often happens in IBD patients (87). Furthermore, pneumonia represents the third most common complication of any surgical procedure, impacting on both morbidity and mortality, prolonging the length of stay, and thus the incidence of further complications (88). Several IBD complications may require a surgical treatment, including strictures, occlusions and fistulas in CD patients and toxic megacolon or poorly controlled disease in UC (89–92). Many studies investigated the impact of pneumonia in a postoperative setting, showing a mortality rate as high as 27%, which was lower in those treated with a laparoscopic approach (93). In addition, low BMI, low nutritional status, and pre-operative hospital stay have been identified as risk factors to develop post-surgical pneumonia in different surgical settings, with higher risk in those undergoing oesophageal surgery or liver transplantation (93). Furthermore, biological therapies may increase the incidence of post-operative infections, especially anti-TNF therapy prior to surgery (94). Nevertheless, at present, no data are available to determine the best moment to discontinue anti-TNF therapy. To our knowledge, no specific studies evaluating the incidence of capsulated infection in IBD patients undergoing a surgical procedure have been performed.

**Figure 1:**
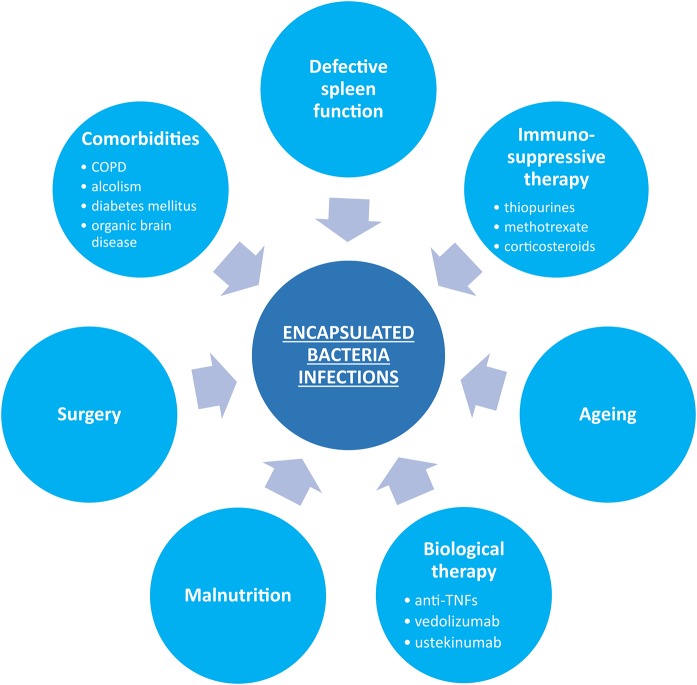
Possible predisposing factors for encapsulated bacterial infections in inflammatory bowel disease.

Figure 1 summarizes factors predisposing to increased infection susceptibility in patients with IBD.

## Role of Vaccination Strategies in Preventing Infections and Mortality

The high clinical impact of risk for capsulate bacterial infections in IBD patients justifies the need for widespread and valid vaccinations strategies in this population, as recommended by current guidelines (5, 18). In particular, anti-pneumococcal, anti-meningococcal, and probably even anti-*Haemophilus influenzae b* vaccines are essential for preventing significant morbidity and mortality in IBD patients, regardless of actual or imminent immunosuppression.

### Anti-pneumococcal Vaccines

Two vaccinations are available against *S. pneumoniae*, a 23-valent polysaccharide vaccine (PPSV23) and a 13-valent conjugate vaccine (PCV13).

PPSV23 contains purified capsular polysaccharides from 23 pneumococcal serotypes, that act as T-cell independent antigens able to stimulate specific IgM production by B cells (95). Due to the exclusively humoral nature of the response induced by PPV23, which does not create an immunologic memory, and the consequent progressive reduction of antibody levels shortly after vaccination, immunization should be repeated every 5 years (96). Immune response to PPSV23 is often inadequate in children <2 years (97), in older adults (98, 99), and in immunosuppressed patients. IBD patients seem to show an adequate response to PPSV23, if they are not on immunosuppressive therapy. Dotan et al. found a significant increase (at least 2-fold) in titer of antibodies to at least 4/14 pneumococcal serotypes in 21/28 (75%) IBD patients, prior to thiopurine therapy start (100); moreover, IBD patients treated with mesalazine were found to have a response to PPSV23 vaccine similar to healthy controls (63). Patients exposure to anti-TNF or to a combination therapy may cause a decrease in vaccination efficacy; conflicting results are available on thiopurine monotherapy. In particular, Fiorino et al. found, after PPSV23 vaccination, a significant lower response in IBD patients receiving infliximab or a combination therapy than in patients taking 5-ASA (57.6 and 62.5% vs. 88.6%), while patients receiving azathioprine showed a normal response rate (78.9%) (64). These results are confirmed by other studies, one on a large cohort of patients affected by CD (65), and one on 45 patients with IBD (101). Other studies on IBD and rheumatologic patients populations showed a stronger influence of immunomodulator therapy (MTX or thiopurines) on reduction of vaccine response rates, compared to anti-TNF alpha alone (101).

PCV13 is a 13-valent conjugate vaccine, in which pneumococcal capsular polysaccharides are conjugated to highly immunogenic cross-reactive material 197 (CRM_197_), a non-toxic diphtheria toxoid protein. The polysaccharide-CRM_197_ complex is bound and internalized by B-cells via polysaccharide-specific IgM and by antigen presenting cells. These cells are able to process and present CRM_197_ protein to type 2 helper T cells. This type of response causes antibody isotype switching and the generation of memory B cells (102). PCV13, due to its higher immunogenicity, is indicated in infants and young children and in adults with immunocompromising conditions (103). In patients affected by CD and not receiving any immunosuppressive drug, PVC13 was shown to induce a higher antibody response to certain serotypes compared to PPSV23 (63); similar results were obtained in a study conducted on a general adult population (104). On the contrary, in IBD (105) and in rheumatologic patients (106) on anti-TNF alpha, or thiopurine, or combination therapy, at least short-term immune response to PCV vaccination resulted to be lower than that to PPSV one, probably due to the drug-induced impairment of T-cell mediated immunity. In order to extend immunological response to a wider range of serotypes, and to boost the response to the serotypes present in both vaccines, a sequential vaccination schedule has been adopted for immunocompromised and for IBD patients, as indicated by the Advisory Committee on Immunization Practice (103) and current guidelines (5, 18) including one dose of PVC13, followed by one dose of PPSV23 after 8 weeks (in immunocompromised patients) or after 1 year (in immunocompetent patients). A second dose of PPSV23 is needed after 5 years and should be regularly repeated in patients older than 65 years old. Efficacy of this approach was evaluated in a rheumatologic setting (107). A recent study by van Aalst et al. (108) studied response rates after sequential vaccination in different groups of IBD patients, including patients in therapy with conventional immunomodulators, with anti-TNF alpha, with combination therapy and not treated by immunosuppressive drugs (controls). Response to vaccination was significantly lower in patients treated with immunosuppressive drugs than in controls (59 vs. 81%), and response impairment was stronger in patients on a combination therapy. These results highlight the necessity for vaccination before commencing immunosuppressive therapy.

### Anti-meningococcal Vaccines

A conjugate vaccine against meningococcal serogroup A, C, W, Y (MenACWY) and a polysaccharide one directed against the same serotypes (MPSV4) are available. Two adsorbed vaccines against serogroup B meningococcus have also been licensed since 2013, MenB-FHbp (three doses at 0, 2, 6 months) and MenB-4C vaccine (two doses at least 1 month apart). Meningococcal vaccines have not been studied in IBD populations, but data are available on general population and on immunosuppressed patients. MenACWY is the most used, and the most effective, anti-meningococcal vaccine directed against serotype A, C, W, Y. It was shown to elicit a significant serological response both in healthy adolescents, in asplenic, and in HIV patients (109). In patients affected by juvenile idiopathic arthritis, adequate antibody titers were found in patients receiving even high doses of immunosuppressive drugs (MTX, infliximab, cyclosporin A). In this group of patients, and in particular in those taking biologics, antibody concentration was lower than non-immunosuppressed patients (110, 111). Also, MenB-FHbp and MenB-4C vaccine showed a good immunogenicity in healthy subjects (112).

### Anti-haemophilus Vaccines

Three monovalent PRP polysaccharide-protein conjugate vaccines are available, namely PRP-OMP, in which purified polyribosylribitol phosphate (PRP) capsular material from Haemophilus influenzae type b (Hib) strains is conjugated with an outer membrane protein complex (OMPC) of the B11 strain of *N. meningitidis* serogroup b, and two PRP-T, in which PRP is conjugated with tetanus toxoid. Different combinate vaccines containing Hib conjugate vaccine have also been licensed. All these vaccines, comprising combinate ones, were shown to induce protective antibody levels in general population, even if with some difference in the timing of antibody response (113). In particular, PRP-OMP is able to induce protective antibody levels after the first dose, while PRP-T confers it after the third dose (after 4 months). Hib vaccine showed a good immunogenicity even in immunocompromised patients, although antibody levels vary with the degree of immunocompetence (113). In a single study conducted on IBD patients, normal response to Hib vaccine was observed both in patients treated with thiopurines and in non-immunosuppressed ones (100).

## Concluding Remarks and Future Directions

Despite the recent advances in the understanding of the mechanisms affecting susceptibility to infections in patients with IBD, many gaps still need to be filled in. In particular, little is known regarding the actual epidemiology of encapsulated bacterial infections in these patients. Also, long term effectiveness of vaccinations is poorly understood, and most of our knowledge derives from studies focusing on patients taking immunosuppressants for other immune-mediated conditions. Nonetheless, definition of clear vaccination strategies is one of the most compelling needs in different settings, including IBD (114, 115). We therefore envisage that future research will focus on this issue.

## Author Contributions

ML, CM, SC, LC, FB, NA, and MV wrote the paper. AD conceived and revised the paper. All the authors approved the final version of this paper.

### Conflict of Interest

The authors declare that the research was conducted in the absence of any commercial or financial relationships that could be construed as a potential conflict of interest.
